# Combining attachment-based family therapy and cognitive behavioral therapy to improve outcomes for adolescents with anxiety

**DOI:** 10.3389/fpsyt.2023.1096291

**Published:** 2023-04-24

**Authors:** Joanna Herres, E. Stephanie Krauthamer Ewing, Suzanne Levy, Torrey A. Creed, Guy S. Diamond

**Affiliations:** ^1^Department of Psychology, The College of New Jersey, Ewing, NJ, United States; ^2^Counseling and Family Therapy Department, Drexel University, Philadelphia, PA, United States; ^3^Perelman School of Medicine, University of Pennsylvania, Philadelphia, PA, United States

**Keywords:** cognitive behavioral therapy, attachment, culturally responsive and trauma-informed practice, parent-adolescent relationship, adolescent anxiety

## Abstract

Increases in adolescent anxiety over the past several years suggest a need for trauma-informed, culturally responsive interventions that help teens cope with environmental stressors like those associated with the COVID-19 pandemic. Although abundant evidence supports the efficacy of cognitive behavioral therapy (CBT) in treating adolescent anxiety, not all teens respond positively to CBT. CBT does not typically include strategies that address important family factors that may be impacting the teen’s functioning, such as the attachment relationship. Attachment-based family therapy (ABFT) addresses the attachment relationship and other factors that contribute to the adolescent’s anxiety and related distress. By enhancing positive parenting behaviors, such as acceptance and validation of the adolescent’s distress and promotion of their autonomy, ABFT sessions may repair the attachment relationship and increase the family’s ability and willingness to engage in CBT tasks aimed at reducing anxiety. This theoretical paper describes the ABFT model and proposes that implementing ABFT sessions prior to CBT could result in better clinical outcomes for adolescents with anxiety disorders by improving the context within which the anxiety symptoms and treatment are experienced. Given that ABFT is sensitive and responsive to family and other contextual factors, adolescents from marginalized communities and those from less individualistic cultures may find the model to be more acceptable and appropriate for addressing factors related to their anxiety. Thus, a combined ABFT+CBT model might result in better outcomes for adolescents who have not historically responded well to CBT alone.

## Introduction

Anxiety among adolescents in the United States is extremely prevalent and has risen in recent years due to escalating environmental stressors related to the COVID-19 pandemic, experiences of racism, and other forms of discrimination, pressures related to social media use, mounting pressures to succeed, and school shootings (1–5). Prevalence rates suggest that an estimated 31.9% of American adolescents are diagnosed with an anxiety disorder at some point in their lives (6), up from a rate of 24% reported in 2016 (7). Chronic stress and anxiety can lead to academic problems, physical problems, and other serious mental health problems, including depression, substance use, and even suicide (8–11).

Given the negative impact of anxiety disorders on functioning, as well as long-term risks of anxiety that can persist into adulthood (12), extensive efforts have been made to identify and disseminate effective treatments. Cognitive behavioral therapy (CBT) has been studied more than any other mode of psychotherapy in the treatment of anxiety (13). CBT includes a fundamental assumption that thoughts, emotions, and behaviors are interconnected, and that psychological distress is maintained by a combination of cognitive factors and learned behavioral responses (14, 15). Per the cognitive model, past experiences influence how a person interprets and reacts to situations. Those thoughts and perceptions vary in how accurate or helpful they are, and more inaccurate and unhelpful cognitions can lead to distressing emotions or problematic responses to those situations. A person with an anxiety disorder might avoid situations they anticipate will be distressing, and that avoidance, in turn, could reinforce the inaccurate or unhelpful beliefs and fuel anxiety, resulting in further distress in the long term. Therefore, an overarching goal in CBT is the promotion of new learning, leading to shifts in one’s beliefs and subsequently in one’s emotions and behaviors.

Cognitive behavioral therapy is considered a first-line approach for treating anxiety disorders among adolescents based on extensive research demonstrating its efficacy (16–19). Despite abundant empirical support for CBT to treat children and adolescents with anxiety, not all children and adolescents show improvement in their anxiety symptoms or functioning. In a meta-analysis of 56 published randomized clinical trials, only 47.6–66.4% of neurotypical clients under 18 years of age showed full recovery in CBT (20). Further, among those who recover, 8% experience a return of their symptoms, with relapse rates even higher in samples of more racially diverse groups (21). These findings suggest that while CBT often has positive and lasting effects for anxious youth,^1^ treatment needs to be improved to facilitate recovery and wellness, particularly for those from historically marginalized populations.

## Parent involvement in CBT

Parents^2^ often play a limited role in CBT for adolescent anxiety, commonly participating in psychoeducation sessions or learning skills alongside their teens to serve as putative coaches outside of session (22). Parents may also be encouraged to help motivate their teens to engage in CBT practices outside of the session, which, in turn, can increase generalization of the teen’s skills outside of the therapy context and speed overall recovery. However, parents of anxious adolescents often engage in behaviors that can lead to greater functional impairment, more severe symptoms, and fewer gains from CBT (23, 24). For example, a parent who anticipates that their teen may experience anxiety or distress in a given situation may suggest things to make the situation “easier” for the teen, like skipping the event, accompanying the teen to provide reassurance, or other well-intended behavior. Paradoxically, these types of strategies, known as accommodation, may increase anxiety by confirming a belief that fears are accurate, and that the avoided situations are either dangerous or unmanageable (25). When parents are involved in CBT, the therapist helps them understand this paradoxical outcome of accommodation and develop alternative responses that encourage their teens to face their fears.

While the empirical support for CBT to reduce anxiety symptoms among children and adolescents is compelling, support for the involvement of parents in CBT is somewhat less so. Although reducing parental accommodation is associated with improvements in anxiety [e.g., (24)], one review of meta-analyses (16) only found an average weighted effect size of *d* = 0.02 across four meta-analyses comparing CBT for child and adolescent anxiety with and without parental involvement, suggesting a negligible difference between the two treatment approaches. The limited evidence for the impact of family involvement in CBT is particularly of note given that family and twin studies provide strong evidence that both environmental and genetic factors play key roles in the etiology of anxiety disorders, with levels of familial aggregation and heritability at 30–60% (26). These influences often come from parents who are likely to experience an anxiety disorder themselves, which can impact how they communicate and interact with their children/teens (27, 28). Involving parents in treatment of adolescent anxiety without addressing the parent/teen relationship might be ineffective because it does not take into account interactional patterns between the adolescent and their parent and other important contextual issues. The relatively weak and inconsistent support for the role of parents in CBT suggests that the model does not target important mechanisms impacting treatment outcome (29, 30).

## Addressing the attachment relationship in treatment of adolescent anxiety

Including parents in CBT might not add significantly to its efficacy because many of the well-established manualized treatments do not adequately address important family factors that contribute to the adolescent’s anxiety. Research suggests that an insecure attachment relationship and family dysfunction contribute to the development and maintenance of anxiety (31–33). The attachment relationship refers to the bond that is formed between a parent and their child early in childhood that is reinforced over time, affecting ongoing interactions between the parent and their child and impacting the child’s emotional adjustment throughout their lifetime (34). Given that CBT for adolescent anxiety disorders targets the adolescent’s thoughts, feelings, and behaviors rather than the parent/adolescent relationship, this treatment modality alone may not address these important interpersonal factors or the impact they have on the adolescent [e.g., (35)]. Benefits of CBT could be potentiated by incorporating treatment strategies that target these interpersonal factors and improve the parent/adolescent attachment relationship.

According to attachment theory (36), when youth are distressed, they are “hard-wired” to seek support and comfort from their parents. In a secure family environment, the youth is confident in their parents’ availability and responsiveness to their needs when stress is activated or when they face a perceived threat. Rather than attempting to suppress or avoid negative emotions resulting from these situations, securely attached youth feel comfortable expressing their emotions, and they are able to tolerate distress rather than avoid it because they know their parents will allow and tolerate their expressions of these emotions and offer support if needed (37). This creates what Bowlby called a “secure base” and “safe haven” (36). Over time, these experiences of protection and safety become internalized as working models (or expectations) about future relationships—related to constructs of core beliefs and intermediate beliefs in a CBT framework (14, 15). If a child is treated well, then they will seek out similar relationships. If they are treated poorly, they develop negative expectations about future relationships. In other words, they develop anxious, avoidant, or disorganized attachment styles to protect themselves from future harm (referred to as compensatory strategies in a CBT frame).

Teens of attentive and sensitive parents are more likely to have a secure attachment style, which is associated with better emotion regulation skills and less anxiety (31). Emotionally attuned parents talk with their teens about their emotions, help them to identify and label their emotions, and validate their emotions. These parenting behaviors result in a secure attachment relationship in which the teen feels safe communicating their emotions to their parents rather than hiding or suppressing them. Parents of securely attached adolescents provide opportunities for their teens to take risks and experience failure while validating the distress they experience from those experiences and expressing confidence in their ability to cope with and adapt to stress. Thus, attachment security helps teens develop effective emotion regulation skills, while promoting their autonomy.

Adolescents with avoidant or anxious attachment styles are not confident that their parents will respond well to their distress (38, 39). Children who do not feel safe expressing their emotional needs to their parents develop patterns of suppressing and avoiding negative emotions as their primary emotional coping strategy (40, 41). Parents of anxious adolescents tend to be high in psychological control (i.e., they are over-controlling of their teen’s emotions) and have difficulty tolerating their teens’ distress (27, 28, 42). They might ignore their teen’s distress, scold them for expressions of distress, or accommodate their anxiety to help them avoid negative emotions. All these strategies fail to teach adolescents how to tolerate or work through difficult emotions. Thus, repairing the parent/teen attachment relationship could improve the teen’s emotion regulation skills and prepare them to engage in activities aimed at reducing their anxiety (31, 43).

## Attachment-based family therapy

Attachment-based family therapy [ABFT; (44)] is a brief, semi-structured family therapy intervention for adolescents that targets the attachment relationship and other relational issues within the family. Research supports the efficacy of ABFT in reducing adolescent attachment anxiety and avoidance, depressive symptoms, and suicidal ideation [see (45) for a review]. Several studies also provide preliminary support for the efficacy of ABFT (in combination with CBT) in reducing adolescent anxiety as well (46, 47). ABFT, combined with CBT, offers the strengths of two evidence-based approaches that together could improve treatment outcomes for anxious adolescents. A combined treatment model that incorporates ABFT strategies could improve the parents’ ability to support the adolescent in navigating and coping with stressors, so that the adolescent is better prepared to engage with CBT tasks directly aimed at reducing their anxiety. Including ABFT in the treatment of adolescent anxiety may be particularly beneficial for adolescents from historically marginalized communities, as ABFT was originally developed with these populations in mind (48–50). Moreover, ABFT’s focus on the family might be more acceptable to those from less individualistic cultures (51, 52). Incorporating ABFT strategies into treatment might better prepare the adolescent for CBT work by improving their relationship with their parents and increasing their confidence in their ability to manage their anxiety.

The ABFT model grew out of the structural family therapy tradition (53) and is informed by more contemporary systemic approaches such as multidimensional family therapy (54) and emotion-focused therapy (55). The model theorizes that the teen’s emotional and behavioral problems are impacted by ruptures in the parent-teen relationship, resulting from major events (e.g., enmeshment, abandonment, neglect, or abuse) or chronic processes (e.g., parenting behaviors high in control and low in warmth). These ruptures may fuel the anxiety directly and/or prevent the teen from discussing their problems with their parents, which promotes emotional dysregulation and avoidance. During ABFT, the therapist guides the teen and their parent(s) (sometimes one parent, sometimes multiple parents) through conversations about times in which the parent failed to provide a secure base for their teen when they were distressed. These conversations provide “corrective attachment experiences” in which the adolescent practices sharing their emotions with their parents while the parents, in turn, provide support and understanding (44). These experiences can be helpful even for securely attached adolescents from relatively high-functioning families because they promote the parents as a secure base so the adolescent is better prepared to practice navigating anxiety-provoking situations.

Attachment-based family therapy helps adolescents meet interpersonal goals that directly relate to their presenting problem. While ABFT for adolescent depression focuses on repairing attachment ruptures (44), ABFT for adolescent anxiety focuses more on promoting independence and competency in order to increase the teen’s courage to face their fears, take risks, make mistakes, and experience discomfort (47). Thus, although the theoretical framework and structure are the same, ABFT for adolescent anxiety focuses on specific aspects of family interactions that contribute to childhood anxiety disorders, including parental beliefs about anxiety, overprotection and accommodation, and psychological control (56–59).

The model uses five distinct treatment tasks that help build the skills needed for the family to engage in difficult conversations about factors that may relate or contribute to the teens’s anxiety. Tasks are not equated with sessions. Instead, a task is a set of procedures, processes, and goals related to resolving or accomplishing specific aims in therapy (e.g., building alliance). These five treatment tasks provide a general scaffold for keeping the therapist focused on core interpersonal dynamics that undermine the adolescent’s trust in the parent and courage to challenge themselves.

Task I establishes an essential foundation for the work: getting family members to agree to initially focus on the attachment relationship and other issues within the family that may be contributing to the adolescent’s anxiety rather than directly on the adolescent’s anxiety and impairment. Topics identified during this task include the teen’s avoidance of their emotions for fear of upsetting their parents and the parents’ over-control of their adolescent’s emotions. Throughout this first task, the family is promoted as “the medicine” to help the adolescent cope with and recover from their anxiety. Tasks II and III consist of building a strong alliance and treatment plan with the adolescent and their parent(s), respectively. In Task II, the therapist meets individually with the adolescent to help them identify and articulate factors that are preventing them from expressing their attachment needs to their parents and motivate them to discuss these with their parents. In Task III, the therapist meets with the parent(s) alone to identify ways in which the parents’ own stressors, anxieties, and intergenerational legacies of attachment affect their parenting style(s) in ways that restrict their adolescent’s autonomy and reinforce their adolescent’s anxiety. The therapist then brings the adolescent and their parent(s) together again in Task IV so that the family can discuss how their interactions contribute to the adolescent’s anxiety. The adolescent shares their thoughts, feelings, and memories about times in which their parent(s) failed to provide a secure base by responding in unsupportive ways to their emotional expressions. Parents acknowledge how their parenting styles may have contributed to their adolescent’s anxiety and related pattern of avoidance, and they offer empathy and support to their child. Although these conversations may not address or resolve all relational problems, this mutually respectful and often emotionally profound dialog serves as a corrective attachment experience, revising the adolescent’s internal working model of self and other. Finally, Task V focuses on the adolescent’s competency and autonomy, as well as the parents’ ability to provide support, advice, and encouragement.

## Combining ABFT and CBT to treat anxious teens

Siqueland et al. (47) demonstrated the feasibility of implementing techniques from both ABFT and CBT in a single-treatment model for adolescents ages 12–18 with anxiety. Though the relatively small sample size meant the study did not have sufficient power to compare differences between the combined ABFT + CBT model and CBT alone, findings provided preliminary evidence that a combined model, implemented in only 16 sessions, can improve anxiety and parental psychological control, as well as other aspects of family functioning. The study also demonstrated the feasibility of training therapists to administer both treatments with fidelity and competence, though therapists with more family therapy training seemed to have an easier time learning and applying ABFT than traditional CBT therapists. Finally, near-perfect retention in the combined ABFT + CBT model suggests that families found the combined model acceptable. In an exit interview, the adolescents and their parents also reported that the family work was the most important or satisfying component of the treatment.

Siqueland et al. (47) suggest beginning treatment of anxious adolescents with ABFT sessions aimed at addressing family beliefs and interactions (sessions 1 through 6) before the adolescent begins CBT work with parental involvement (sessions 6 through 12). Given findings that suggest that improving the attachment relationship may help adolescents with avoidance tendencies approach and better tolerate distress (60), ABFT sessions might improve the adolescent’s confidence in their ability to approach and confront threatening and anxiety-provoking situations. Thus, providing ABFT sessions prior to CBT might better prepare the teen and increase their willingness to engage in exposure exercises that directly address maladaptive cognitions and behavioral responses contributing to the teen’s anxiety symptoms. Implementing ABFT strategies early could also help reduce the likelihood that the parents will accommodate their teens avoidance. Accordingly, ABFT sessions could lay the groundwork necessary for the adolescent to engage in CBT work.

## Implementation of ABFT with diverse adolescents

Attachment-based family therapy was developed to respond to the needs of diverse adolescents. Thus, combining ABFT and CBT might result in improved outcomes for adolescents with anxiety from marginalized communities. Prior studies demonstrate that ABFT can be effectively implemented with teens and families from a variety of demographic backgrounds (61). For example, two randomized controlled clinical trials in the United States were conducted with samples in which a large percentage of teens (74% in the first trial and 49% in the second trial) identified as Black/African American (48, 62, 63), a group in the United States that has been historically marginalized and very likely to contend with race/ethnicity-related stress and trauma. Experiences of racial trauma and minority stress can fuel symptoms (64), and attending to these factors is associated with improved implementation outcomes in CBT, such as the family’s level of satisfaction with treatment (65, 66). While African Americans have historically shown great resilience in the face of adversity, recent killings by police of unarmed Black people, including Black teens, have exposed this population to new racial trauma, increasing their risk of anxiety (67). ABFT offers a framework for addressing racial trauma when working with teens and their parents. Addressing these contextual issues in ABFT sessions might reduce parental accommodation and better prepare the teen to engage in CBT sessions more directly aimed at addressing their anxiety.

One case study highlights the ways in which discussions about culture, racism, and identity development can surface during a course of ABFT (68). In this case, once the teen and her mother had explored and addressed barriers in the quality of their relationship (Tasks 1–4), the teen was able to more openly discuss and turn to her mother for guidance and support around her distressing experiences with racism, sexism, and colorism (prejudice against darker skin tone). The family was able to acknowledge and process the ways in which the parents’ anxiety and experiences of racism also impacted the teen, the mother’s ability to support the teen, and the positive and negative socialization messages she communicated to her teen. Thus, in Task V (autonomy building) specifically, the ABFT therapist works with the family around issues related to oppression and discrimination, if relevant (69–72).

Evidence of the feasibility and acceptability of ABFT with families in the child welfare system demonstrates that the treatment model also works well with families with complex histories and chronic, cross-generationally dysfunctional family dynamics (73). Additionally, preliminary studies with teens who identify as sexual and gender minorities (SGMs) provide data that demonstrate improved outcomes, including increased parental acceptance, decreased parental rejection, and decreased attachment avoidance, as well as feasibility and acceptability, as rated by teens, families, and therapists (50). ABFT provides opportunities for the therapist to work with the parents of teens who identify as SGMs to process any fears and worries they may have for their teen and themselves, possibly grieve lost social support, reduce rejecting behavior, and increase accepting behaviors, if possible (50, 61, 74).

Attachment-based family therapy may be associated with improved clinical outcomes among diverse adolescents because ABFT training offers therapist-specific guidance for addressing contextual factors like racism and minority stress. Training workshops outline specific points during therapy when the therapist could address these issues. Further, during supervision, the supervisor engages the ABFT therapist in discussions about how they talk in therapy about their own and their client’s identities and how they converse about issues like discrimination, racism, and oppression. The supervisor helps the therapist work through uncomfortable moments they encounter with clients related to identity and process ways to navigate ruptures with clients when they occur. Therapists are encouraged to use what they have learned from the families about their identities and experiences to help inform their conceptualization of the presenting problem and the attachment relationship, potential ruptures that may exist related to issues of identity, how issues of identity are related to the young person’s presenting problem, and how to help the family make changes in ways they engage with one another. Thus, the ABFT therapist is taught to approach their work with families with cultural humility by taking into consideration a variety of aspects about themselves and their clients (e.g., social class, gender, race, ethnicity, sexual orientation, religion, and disability status) and striving to understand each family member’s unique experience and how it informs their beliefs and values. It is essential to understand how the intersectionality of identity and marginalized status impacts the adolescent’s experience of anxiety, the parent’s beliefs about, experience of, and response to their teen’s anxiety, and how parents might unintentionally contribute to their teen’s anxiety. This attention to cultural humility in ABFT may be important in priming adolescents to effectively utilize CBT skills.

## Discussion

While parents are often involved in CBT for youth anxiety, parental involvement does not offer incremental benefits over individual child/teen-focused therapy (16). Although parents may be involved in CBT, the model does not address mechanisms related to the attachment relationship that may be impacting symptoms and interfering in the adolescent’s recovery (30). Research and theory on risk of adolescent anxiety suggests a need to address the attachment relationship in treatment [e.g., (31)]. ABFT addresses important family issues and, if implemented prior to CBT, might potentiate benefits for adolescents with anxiety. Combining ABFT and CBT might also result in better outcomes than CBT alone, because ABFT offers a culturally responsive, trauma-informed approach (66, 75). Thus, adolescents from marginalized backgrounds might find CBT more acceptable and appropriate for addressing their concerns if it is combined with ABFT.

Although several studies support the use of ABFT to treat adolescent anxiety (46, 47), a larger trial is needed to compare clinical outcomes of a combined model to either treatment alone. Potential outcomes include reduction in anxiety symptoms, acceptability and feasibility of the combined treatment, and treatment satisfaction. Research should also examine the need for specialized training in implementing a combined model, therapists’ comfort in implementing a combined model, and therapist adherence to each model. This research might consider the benefits of a team approach in which an ABFT therapist and a CBT therapist take turns providing the different treatment components. Another potential challenge might be implementing elements of both treatments in a relatively brief amount of time. However, Siqueland et al. (47) demonstrated the feasibility of implementing a combined model in only 16 sessions, and based on this work, they suggest that a combined model could be implemented in as few as 12 sessions. Another area for future research would be to explore potential mechanisms of change. This research could test whether improvements in the attachment relationship and related factors (e.g., parental psychological control) mediate treatment outcome. Additionally, future research should consider whether all adolescents with anxiety would benefit from the combined model or whether the decision to incorporate ABFT into treatment would depend on whether the adolescent is experiencing problems in their attachment relationship. It is possible that only the most dysfunctional families would benefit from the family work. Finally, future studies should examine whether anxious adolescents from marginalized groups would respond better to a combined ABFT + CBT model vs. either treatment alone.

## Data availability statement

The original contributions presented in the study are included in the article/supplementary material, further inquiries can be directed to the corresponding author.

## Author contributions

JH, EK, SL, TC, and GD contributed to conceptualization, writing, and revision. All authors contributed to the article and approved the submitted version.

## Conflict of interest

GD and SL receive federal and private foundation grant funding to study ABFT, royalty from the ABFT book grant, and salary support from money generated by the ABFT training center at Drexel.

The remaining authors declare that the research was conducted in the absence of any commercial or financial relationships that could be construed as a potential conflict of interest.

## Publisher’s note

All claims expressed in this article are solely those of the authors and do not necessarily represent those of their affiliated organizations, or those of the publisher, the editors and the reviewers. Any product that may be evaluated in this article, or claim that may be made by its manufacturer, is not guaranteed or endorsed by the publisher.

## Footnotes

^1^We use the term "youth" to refer to anyone under the age of 18.

^2^Families come in many constellations and primary caregiving responsibilities may be provided by various people including parents, step-parents, grandparents, a single parent, and others. We use the term "parents" herein for brevity to refer to whoever provides parenting to a child.
